# Healing effects of *Musa sapientum* var. *paradisiaca* in diabetic rats with co-occurring gastric ulcer: cytokines and growth factor by PCR amplification

**DOI:** 10.1186/1472-6882-13-305

**Published:** 2013-11-05

**Authors:** Mohan Kumar, Manish Kumar Gautam, Amit Singh, Raj Kumar Goel

**Affiliations:** 1 Department of Pharmacology, Institute of Medical Science, Banaras Hindu University, Varanasi 221005, India

**Keywords:** *Musa sapientum*, Diabetes, Ulcer healing, TNF-α, IL- 1β, TGF-α

## Abstract

**Background:**

The present study evaluates the effects of extract of *Musa sapientum* fruit (MSE) on ulcer index, blood glucose level and gastric mucosal cytokines, TNF-α and IL-1β and growth factor, TGF-α (affected in diabetes and chronic ulcer) in acetic acid (AA)-induced gastric ulcer (GU) in diabetic (DR) rat.

**Methods:**

MSE (100 mg/kg, oral), omeprazole (OMZ, 2.0 mg/kg, oral), insulin (INS, 4 U/kg, sc) or pentoxyphylline (PTX, 10 mg/kg, oral) were given once daily for 10 days in 14 days post-streptozotocin (60 mg/kg, intraperitoneal)-induced diabetic rats while, the normal/diabetic rats received CMC for the same period after induction of GU with AA. Ulcer index was calculated based upon the product of length and width (mm^2^/rat) of ulcers while, TNF-α, IL-1β and TGF-α were estimated in the gastric mucosal homogenate from the intact/ulcer region. Phytochemical screening and HPTLC analysis of MSE was done following standard procedures.

**Results:**

An increase in ulcer index, TNF-α and IL-1β were observed in normal (NR)-AA rat compared to NR-normal saline rat, which were further increased in DR-AA rat while, treatments of DR-AA rat with MSE, OMZ, INS and PTX reversed them, more so with MSE and PTX. Significant increase in TGF-α was found in NR-AA rat which did not increase further in DR-AA rat. MSE and PTX tended to increase while, OMZ and INS showed little or no effect on TGF-α in AA-DR rat. Phytochemical screening of MSE showed the presence of saponins, flavonoids, glycosides, steroids and alkaloids and HPTLC analysis indicated the presence of eight active compounds.

**Conclusion:**

MSE showed antidiabetic and better ulcer healing effects compared with OMZ (antiulcer) or INS (antidiabetic) in diabetic rat and could be more effective in diabetes with concurrent gastric ulcer.

## Background

Extensive investigations regarding anti-ulcerogenic and ulcer healing activities of plantain banana have been carried out in our laboratory for the past 30 years. Anti-ulcerogenic activity of dried powder of plantain banana fruit pulp (*Musa sapientum* var. *paradisiaca*, MS) against various experimental gastro-duodenal have been reported from our laboratory and elsewhere. Ulcer protective effects of plantain banana was reported due to increase in the defensive factors viz. increase in mucin secretion, mucosal glycoproteins, accumulation of prostaglandins E and I_2_, life span and cell proliferation rather than affecting the offensive acid-pepsin secretion [1-6]. Recently we reported ulcer protective, plantain banana enhancing antioxidants, superoxide dismutase (SOD), catalase (CAT) and glutathione peroxidase (GSH) and decreasing the free radicals, lipid peroxidation (LPO) and nitric oxide (NO) levels in the gastric mucosal homogenates without any anti-*H. pylori* activity *in vitro*[7]. Several reports indicate that diabetes mellitus (DM) increases the mucosal susceptibility to ulcerogenic stimuli and predisposition to gastric ulceration and impaired healing [8,9]. Our earlier reported work on streptozotocin-induced diabetes with co-occurring gastric ulceration did indicate the involvement of both offensive acid-pepsin secretion and free radical generation and defensive factors like mucus secretion, mucosal glycoproteins, life span of mucosal cells and antioxidant status of gastric mucosa which were increased and decreased respectively [10,11].

Pro-inflammatory cytokines like tumor necrosis factor-α (TNF-α), Interleukin-1β (IL-1β), Interleukin-6 (IL-6) and growth factors including insulin like growth factor-1 (IGF-1) play an important role in the pathogenesis of chronic diseases like autoimmune diabetes and gastric ulcers [12,13]. Growth factors like vascular endothelial growth factor (VEGF), basic fibroblast growth factor, transforming growth factor-α (TGF-α) are having major role in tissue healing processes including that of gastric ulcers [9,14]. Recently, we reported the healing effects of ethanolic extract of dried fruit pulp of MS (MSE) on acetic acid induced gastric ulcer in normal (NR) rat and found the role of cytokines, TNF-α, IL-1β and growth factor, TGF-α in healing ulcer [15].

Hypoglycemic activity has been reported from green fruit of plantain banana. Further, leucocyanidin, a flavonoid, was isolated from plantain banana and its dimethoxy derivative, leucocyanidin 3-O-beta-D-galactosyl cellobioside, demonstrated hypoglycemic effect in experimental animal [16,17]. Therefore, the present study was extended to evaluate the healing effects of MSE (test drug) in acetic acid-induced gastric ulcer in diabetic (DR-AA) rat and its effects on TNF-α, IL-1β and TGF-α. The effects of MSE on gastric ulcer, TNF-α, IL-1β and TGF-α were compared with anti-ulcer drugs, omeprazole (OMZ, proton pump inhibitor) and pentoxyphylline (PTX, an inhibitor of TNF-α_,_ a prominent inflammatory marker) and hypoglycemic drug, insulin (positive controls) and CMC-treated (negative control) in DR-AA rat.

## Methods

### Animals

Inbred Charles-Foster albino rats (200–250 g) of either sex were obtained from the central animal house of Institute of Medical Sciences, Banaras Hindu University, Varanasi. They were kept in the departmental animal house at 26 ± 2°C and relative humidity 44-56%, light and dark cycles of 10 and 14 hours respectively for 1 week before and during the experiments. Animals were provided with standard rodent pellet diet (Pashu Aahar, Ramnagar). Food was withdrawn 18–24 hours before the experiment though water was allowed *ad libitum*. Animals were divided into seven groups containing 6 animals each. First two groups were having normal rats while 3^rd^ to seventh groups had diabetic rats. Gastric ulcers were produced with acetic acid in all the groups except 1^st^ group (NR + NS), where NS was applied to the stomach wall instead of acetic acid. 1^st^ to 3^rd^ groups received CMC orally while 4^th^ to 7^th^ groups received ethanolic extract of fruit pulp of *Musa sapientum* (MSE), omeprazole (OMZ), insulin (INS) and pentoxyphylline (PTX) for 10 days respectively after induction of GU with acetic acid. ‘Principles of laboratory animal care’ (NIH publication no. 82–23, revised 1985) guidelines were followed. Approval from the Institutional Animal Ethical Committee was taken prior to the experimental work (Dean 542/02/ab/CPCSEA dated 23.8.2002).

### Collection and preparation of extract

Big sized, unripe, green plantain banana fruits (*Musa sapientum Linn*. var. *paradisiaca,* MS) collected during the month of November were obtained from the local market and identified by Prof. V.K. Joshi, Department of Dravyaguna, Faculty of Ayurveda, IMS, BHU. A Sample specimen (MS-09/231) was preserved in the Department of Dravyaguna, IMS, BHU, Varanasi. The skin of the fruit was peeled off and the pulp was cut into small pieces and dried at room temperature. After shade drying powder of the pulp was prepared from the small pieces and used for extraction. The ethanolic extract (MSE) was prepared by adding 1 liter of ethanol twice, at an interval of two days. The ethanol containing extract so obtained each time was mixed and later dried in vacuum drier. MSE was stored at −20°C until further use. The yield of the extract was 1.6% (w/w).

### Phytochemical study

Chemical constituents like saponins, flovonoids, glycosides, steroids and alkaloids were estimated in the MSE following the standard procedures [18].

### HPTLC finger printing evaluation

MSE was subjected to HPTLC (CAMAG TLC system) finger printing analysis. The extracts were diluted in order to get a concentration of 1 μg/μl and applied on pre- coated silica gel G60 F_254_ plates (10x10, 2 mm thickness, E. Merck) by using LINOMAT IV spotter. The plate was then developed in CAMAG flat bottom chamber under conditions of partial or complete saturation of the tank atmosphere with solvent vapors. The solvent system used was chloroform: methanol: formic acid (9:1:0.5). The plate was carefully dried and sprayed with anisaldehyde-sulphuric acid followed by heating at 110°C for 5–10 min. The detection of spots was made by using Hg lamp at 254 nm and images were recorded by using CAMAG Reprostar 3. The plate was scanned by using CAMAG TLC scanner 3 with WinCATS evaluation software.

### Drugs and chemicals

Streptozotocin (St. Louis, MO, USA), omeprazole (Cipla, Mumbai, India) and pentoxyphylline (Abbott, Bangalore, India) were used in the present study.

### Induction of diabetes

Diabetes was induced in adult rats (200–250 g) by a single intraperitoneal injection of streptozotocin (STZ, 60 mg/kg) dissolved in citrate buffer (pH 4.5) and fed normally thereafter [19] while, the control rats received citrate buffer only. Blood samples were collected from the retro-orbital plexus of the rats and serum was separated by centrifugation at 3000 rpm for 3 minutes. The blood glucose levels were estimated in the unhemolysed serum by Glucose oxidase-peroxidase (GOD-POD) method using glucose diagnostic kit (Ranbaxy Laboratories Ltd, India). Blood glucose level (BGL) was estimated after 48 hours and two weeks of streptozotocin administration and rats showing BGL above 250 mg/dL under non fasting conditions were selected for gastric ulcer study. Body weight changes have been observed before (0 day), after 14 days post diabetic or normal rats and on the 10^th^ day of AA-induced GU after CMC/MSE/OMZ/INS/PTX treatments (24^th^ day) in normal and diabetic rats.

### Acetic acid- induced gastric ulcer in diabetic rats and treatment protocol

Diabetic rats were anaesthetized with pentobarbitone (35 mg/kg, intraperitoneal). The abdomen was cut open with an incision and the stomach was visualized. A cylindrical glass tube of 6 mm in diameter was tightly placed upon the anterior serosal surface of the glandular portion of stomach 1 cm away from the pyloric end. 100% acetic acid (0.06 ml/animal) was instilled into the tube and allowed to remain 60 seconds on the gastric wall. After removal of the acid solution, the abdomen was closed in two layers and animals were caged and fed normally. MSE (100 mg/kg, oral), OMZ (2.0 mg/kg, oral), INS (4U/kg, subcutaneous) and PTX (10 mg/kg, oral) were administered, once daily, first dose being given 4 hours after the application of acetic acid on day 1 and then continued up to 10 days. The animals were put on fast for 24 hours after the last dose of test drugs on 10^th^ day and sacrificed by euthanasia on 11^th^ day of experiment to assess the gastric ulcer size and healing. Ulcer index was calculated based upon the product of length and width (mm^2^/rat) of ulcers [20].

### TNF-α, IL-1β and TGF-α estimations

TNF-α, IL-1β and TGF-α were estimated in intact gastric mucosa and from gastric mucosa of diabetic rats exposed to acetic acid with or without different drug treatments. Mucosa from the glandular portion of stomach were scraped off from the ulcerated/intact area using sterilized glass slides and stored at −80°C until analysis. Total RNA was extracted from mucosa samples by the method of Chomczynski and Sacchi (1987) [21] using the extraction kit from Bangalore Genei, India. The isolation of RNA was quantified and 5 μg of total RNA was used to prepare cDNA. The PCR mixture was amplified in a DNA thermal cycler with the specifications described in materials and methods. Thus β-actin, IL-1β, TNF-α and TGF-α were estimated. The results have been expressed as ratio of the cytokines/growth factor to β-actin in the different treated groups.

### Total RNA isolation from gastric mucosa

Approximately 100 mg of gastric mucosal scrapping from the glandular region (site of ulcer area) near to the ulcerated area was taken into DEPC treated tube and homogenized with 1 ml of denaturing solution. To this 1 ml of water saturated phenol followed by 200 μl of chloroform – isoamyl alcohol mixture (freshly prepared in the ratio of 49:1) was added and mixed thoroughly. The mixture was incubated in ice for 15 minutes and centrifuged at 10000 rpm for 20 minutes at 4°C. The upper layer was carefully removed in to another DEPC treated tube and 1 ml of 100% isopropanol was added and kept at −20°C for 30 minutes to precipitate the RNA. Again the mixture is centrifuged at 10000 rpm for 20 minutes at 4°C and the supernatant was discarded. The pellet was resuspended in 0.3 ml of denaturing solution and precipitated by adding equal amount of 100% isopropanol. This mixture was kept at −20°C for 30 minutes and centrifuged at 10000 rpm for 20 minutes at 4°C. The supernatant was discarded and the pellet was washed with 75% ethanol to remove any residual amounts of guanidine for 15 minutes. Again the content was centrifuged for 20 minutes at 4°C at 10000 rpm. The supernatant was discarded and the pellet containing RNA was dissolved in 100 – 200 μl of DEPC treated water and incubated at 55°C for 10–15 minutes. This total RNA was stored at −70°C until further use. The total RNA was quantified by taking the absorbance at 260 nm (A_260_) and by using the following formula i.e. concentration of RNA sample = 40 × A_260_ × dilution factor i.e. an absorbance of 1 unit at A_260_ corresponds to 40 μg of RNA per ml (This relation is valid only for measurements in water).

### First strand cDNA synthesis

5 μg of total RNA was taken from each sample in a sterile RNA free (DEPC treated) tube and sterile water was added to make up the final volume to 9 μl. To this 1 μl of Oligo (dT)_18_ primer was added and placed at 65°C for 10 minutes and then at room temperature for 2 minutes. To this 1 μl of RNAs inhibitor, 1 μl of 0.1 M DTT, 4 μl of RT buffer (5x), 2.0 μl of 30 mM dNTP mix, 0.5 μl of Reverse Transcriptase and 1 μl of sterile water were added and mixed well. The mixture was kept at 42°C for 1 hour and incubated at 95°C for 2 minutes and kept in ice quickly. This cDNA product can be directly used for PCR amplification or otherwise store at −20°C for further use.

### Polymerase chain reaction (PCR) amplification

The resultant cDNA (2 μl) was amplified in a 50 μl reaction volume containing 2 U of Taq polymerase, dNTP 1 μl (30 mM), 5 μl of 10 × PCR buffer and specific primers (forward and reverse) were used at a final concentration of 1 mM. The polymerase chain reaction mixture was amplified in a DNA thermal cycler (MJ Research) and the incubation and thermal cycling conditions were as followed: denaturation at 94°C for 2 minutes, annealing at 60, 66 and 62°C for β actin, TGF – α, IL – β and TNF – α respectively and extension at 72°C for 50 seconds and final extension for 2 minutes. The number of cycles is 30 for all the reactions. The nucleotide sequence of the primers were as followed: β actin, sense 5′-TTG TAA CCA ACT GGG ACG ATA TGG – 3′; antisense 3′ – GAT CTT GAT GGT GCT AGG – 5′ (764 bp); TGF – α sense 5′ – ATG GTC CCC GCG GCC GGA CA- 3′; antisense 3′ – GAC CAC TGT CTC AGA GTG GCA GCA AGG CAG TCC TTC TTT – 5′ (476 bp); IL – 1β sense 5′- GCT ACC TAT GTC TTG CCC GT -3′; antisense 3′ – GAC CAT TGC TGT TTC CTA GG – 5′ (543 bp); TNF – α sense 5′ – TAC TGA ACT TCG GGG TGA TTG GTC C – 3′; antisense 3′ – CAG CCT TGT CCC TTG AAG AGA ACC – 5′ (295 bp). The primers were selected based on the sequences published in earlier study [9] were synthesized by Bangalore Genei, Bangalore, India.

Polymerase chain reaction products were detected by electrophoresis on a 1.5% agarose gel containing ethidium bromide. Location of predicted product was confirmed by using 300-bp DNA ladder (Bangalore Genei, India) as a standard size marker. The gel was then photographed by under UV transillumination. The density of the PCR products was measured by using alpha imager software. The signal for PCR products were standardized against that of β-actin mRNA from each sample and the results were expressed as PCR product/β actin mRNA ratio.

### Statistical analysis

Unpaired Student‘t’ test was used for Statistical comparison between two groups. Comparisons involving more than two groups were performed using one way analysis of variance (ANOVA) and for multiple comparisons versus control group was done by Dunnett’s test.

## Results

### Phytochemical study

**Figure 1:**
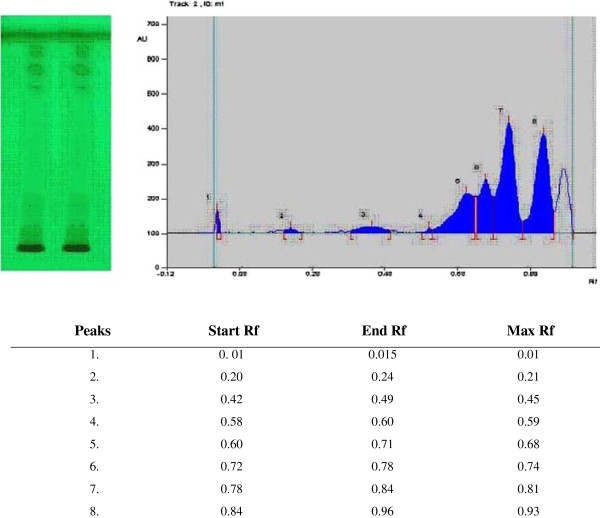
The finger printing of HPTLC chromatogram of MSE.

Preliminary phytochemical screening of MSE showed the presence of saponins, flovonoids, glycosides, steroids and alkaloids by chemical testing. The finger printing of High-Performance Thin-Layer Chromatography (HPTLC) chromatogram showed the presence of at least eight active compounds (Figure 1). The analysis of retention factor and colors of the resolved bands at 254 nm at R_f_ 0.71 in MSE could be a flavonoid, leucocyanidin as earlier reported by Prabha and associates [17] where they have shown the similar result with the methanolic extract of *Musa sapientum* fruit.

### Blood glucose study

Normal (NR) + NS (98.7 ± 6.0 mg/dL) and NR + AA (106.3 ± 5.8 mg/dL) rats showed little or no change in blood glucose level (BGL). A dose-dependent anti-hyperglycemic study was undertaken with 50, 100 and 200 mg/kg of MSE administered for 10 days to diabetic rats and a decrease of 11.0, 17.0 and 19.9% (*P* < 0.3 to *P* < 0.1, n = 6) in BGL was observed (BGL- 297.6 ± 21.2 mg/dL). MSE (100 mg/kg) tended to decrease (17.0% decrease, *P* < 0.1) but INS decreased the BGL (61.4% decrease, *P* < 0.05) while, OMZ and PTX showed little or no change in BGL compared with DR + AA group. However, dose of 100 mg/kg was selected for further study as this dose was earlier found effective in healing acetic acid-induced gastric ulcer in normal rats^15^.

### Body weight changes

The mean ± SE body weights of rats in all the 7 groups studied at 0 day showed a range of 201.3 ± 3.4 to 205.4 ± 2.9 g/rat at the start of experiment. 1^st^ and 2^nd^ were normal rat groups while 3^rd^ to 7^th^ was diabetic rat groups. Increase in body weight was observed in 1^st^ (8.9%, P < 0.05) and 2^nd^ (5.6%) groups while the diabetic rats of 3^rd^ to 7^th^ group showed decrease in body weight by 8.7 to 11.3% (P < 0.05 to P < 0.01) respectively on 14^th^ post normal/diabetic day compared with their respective 0 day value. There was significant gain in weight after treatments with MSE (9.7%, P < 0.05) and INS (15.3%, P < 0.01) while, little increase in body weight was observed with OMZ (3.4%) and PTX (4.7%) treatments compared to their respective 14 days value. However, diabetic control rats did not show any increase in their body weight from its 14 day value.

### Gastric ulcer healing

**Figure 2:**
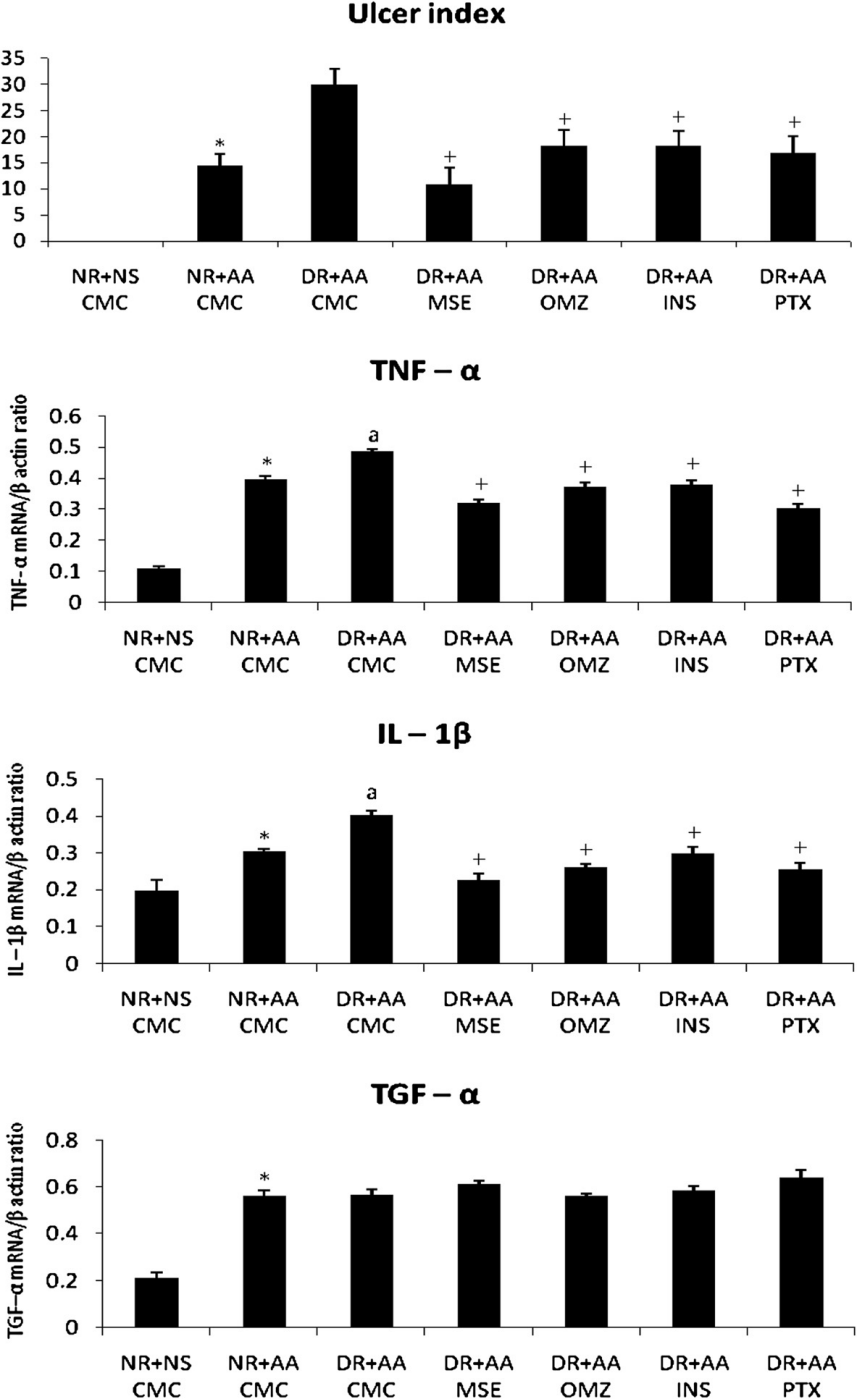
**Effects of ethanolic extract of fruit pulp of *Musa sapientum *(MSE, 100 mg/kg × 10 days), omeprazole (OMZ, 2 mg/kg × 10 days), insulin (INS, 4U/kg × 10 days) and pentoxyphylline (PTX, 10 mg/kg × 10 days) on rat gastric ulcer index and gastric mucosal cytokines, tumor necrosis factor-α (TNF- α) and interleukin-1β (IL-1β) and transforming growth factor- α (TGF- α) as ratio to β actin in acetic acid (AA)-induced gastric ulcer in normal (NR) and diabetic (DR) rats.** Results are mean + SEM, 4 animals in cytokines and growth factor study and 6 animals in gastric ulcer study. CMC, MSE, OMZ and PTX were administered orally while; INS was given by subcutaneous route. ^*^P <0.05 compared to NS group, ^a^*P* <0.05 compared to NR + AA group and ^+^*P* <0.05 compared to DR + AA group (Statistical analysis was done by one way ANOVA followed by Dunnett’s test for multiple comparisons).

NR + AA rat showed increase in ulcer index (UI-14.3 ± 2.3, *P* <0.05) compared to NR + NS (No ulcer) rat while, DR + AA rat showed further increase in ulcer index (108.4% increase, *P* < 0.05) compared to NR + AA rat indicating increased propensity to ulceration and delay in ulcer healing. DR + AA rats, treated with OMZ, INS and PTX showed decrease in ulcer index by 39.3, 38.6 and 43.3% respectively (near to NR + AA level) while, MSE showed decrease by 63.8%, less than the value of NR + AA rats indicating better healing by MSE compared to OMZ, INS or PTX (Figure 2).

### TNF-α, IL-1β and TGF-α

**Figure 3:**
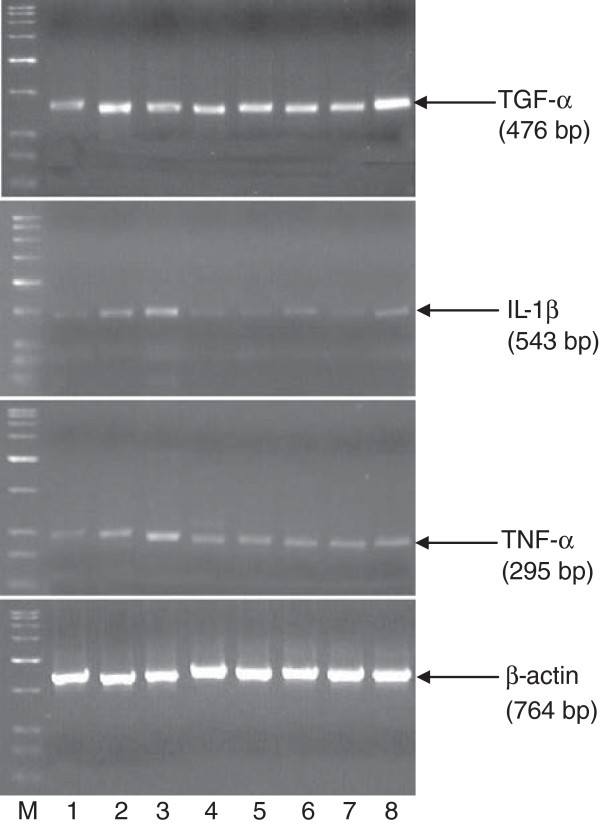
**Effect of MSE, OMZ, INS and PTX on gastric mucosal TNF-α, IL-1β and TGF-α levels in diabetic rats (DR) (RT-PCR method).** Lane M: Marker. Lane 1: NR + NS. Lane 2: NR + AA. Lane 3: DR + AA. Lane 5: DR + AA + MSE. Lane 6: DR + AA + OMZ. Lane 7: DR + AA + INS. Lane 8: DR + AA + PTX.

The results of TNF-α, IL-1β and TGF-α have been expressed as ratio of the cytokines/growth factor to β-actin (Figures 2 and 3).

### TNF-α and IL-1β

NR + AA rats showed increase in the gastric mucosal TNF-α by 265.1% (*P* < 0.05) and IL-1β by 52.8% (*P* < 0.05) compared to NR + NS (intact mucosa) rats. DR + AA rats showed further increase in the levels of IL-1β and TNF-α by 345.0 and 102.5% respectively (*P* < 0.05) compared to respective NR + NS rats and by 21.9 and 32.6% increase (*P* < 0.05) compared to respective NR + AA rats. Treatments of DR + AA rats with hypoglycemic drug, INS and anti ulcer drugs, MSE and OMZ and PTX reversed the levels of IL-1β and TNF-α near to the NR + AA level. However, both MSE and PTX showed better decreasing effects on TNF-α and IL-1β compared to OMZ or INS (Figures 2 & 3).

### TGF-α

NR + AA rats showed significant increase in gastric mucosal TGF-α (165.1% increase, *P* < 0.05) compared to NR + NS rats. However, TGF-α was not further increased in DR + AA rats compared to NR + AA group. Treatments of DR + AA rats with OMZ and INS did not increase the TGF-α level further while, MSE and PTX tended to increase TGF-α (Figures 2 & 3).

## Discussion

Experimental diabetic rats have shown increased propensity to gastric ulceration and impaired healing of gastric lesions [8,9] and this aggravation of ulcer/delayed healing were reversed by agents correcting blood sugar level [22]. Non-insulin dependent diabetes mellitus (NIDDM) increased the propensity to gastric ulceration by enhancing offensive, acid-pepsin secretion and gastric mucosal free radicals, lipid peroxidation (LPO), nitric oxide (NO) and decreasing mucosal glycoprotein and antioxidants, superoxide dismutase (SOD) levels [10].

Streptozotocin (STZ) is widely used to induce experimental diabetes in animals and its cytotoxic action is mediated by reactive oxygen species. STZ seems to be more specific as there is uptake by beta cell specific GLUT2, but not by other glucose transporters and have less side effects. It causes activation of poly ADP-ribosylation, which leads to formation of superoxide radicals and as a result, β cells undergo the destruction by necrosis [23]. The mechanism underlying the increased susceptibility of gastric mucosa in diabetic animals to damage is multifactorial and includes the attenuation of angiogenesis as well as the increased production of proinflammatory cytokines, TNF-α and IL-1β which result in sustained inflammatory reaction and delaying healing process at the ulcer area [9]. The major mechanism by which cytokines exert their deleterious influences on ulcer healing may involve the inhibition of growth factors responsible for mucosal regeneration and recovery from the damage [24]. It has also been reported that TNF-α augments the production of IL-1β and other cytokines, leading to neutrophil accumulation [25] and enhanced production of ROS in the gastric mucosa exposed to ischemia-reperfusion. Pentoxifylline, an inhibitor of TNF-α has been reported to accelerate healing of the acetic acid-induced gastric ulcers in rats possibly via inhibition of TNF-α production [13]. It has been reported that the enhancement of cell proliferation during ulcer healing may be mediated by increased release of EGF and TGF-α. It has also been reported that despite the increase in TGF-α level in the diabetes rats with co-occurring gastric ulceration the healing was delayed due to unknown mechanism/s. It was proposed that the hyperglycemia in diabetic animals could induce some structural modifications of TGF-α or its receptors in the ulcer area, leading to the dysfunction of this growth factor during diabetes mellitus [9,26].

Acetic acid (AA)-induced ulcer in rat were reported to have close resemblance with clinical ulcers in location, chronicity and severity and serves as the most reliable model to study ulcer healing process [20]. In the present study, we have observed increased levels of proinflammatory cytokines TNF-α and IL-1β in the gastric mucosa of normal rats treated with acetic acid. Diabetic rats showed a further increase in their level compared to normal rats when subjected to acetic acid-induced gastric ulceration. The above observation has been in confirmation with the earlier reported works [13,27,28]. MSE was reported to reverse the increase of TNF-α and IL-1β in GU induced by AA in normal rats and promoted ulcer healing by its various effects on gastric mucosal defensive factors including enhanced cell proliferation and antioxidants and reducing free radicals levels [7,15]. The treatment with insulin reversed the impaired ulcer healing in diabetic animals, mainly due to the normalization of hyperglycemia, but not to the direct effect of insulin on the ulcer healing because insulin administered to non-diabetic rats did not show any effect in ulcer healing [27]. This showed that antidiabetic drugs were effective by correcting the increased glucose level thereby reducing the levels of these cytokines whereas, anti ulcer drug (omeprazole) was enhancing the ulcer healing and reducing their level [15]. TNF-α was reported to stimulate the up regulation of IL-1β levels, so by controlling the TNF-α expression the IL-1β expression could be controlled. This may be the mechanism by which pentoxifylline decreases both the cytokines levels [29].

The present work showed increase in TGF-α level in rats treated with acetic acid. However, no further increase in TGF-α level was found in diabetic rats and this observation is consistent with previous observations showing the importance of this growth factor in the process of ulcer healing. However, the fact that a delay of healing of the gastric ulcers in the diabetic state, despite increased expression of TGF-α, indicates that the healing effects of this growth factor may be attenuated by some unknown mechanism. This militates against the major role of this factor in the observed delay in healing of gastric ulcers under diabetic conditions. One possibility is that the hyperglycemia in diabetic animals could induce some structural modifications of TGF-α or its receptors or decrease the availability of essential ingredients required for normal healing in the ulcer area, leading to the dysfunction of this growth factor during diabetes mellitus. Further studies are needed to answer the question why increased expression of TGF-α does not contribute to the release of a functionally active growth factor [9]. It was also reported that the increased TGF-α level might increase the cell proliferation [30]. MSE showed a tendency to increase TGF-α level in diabetic rats. This may be better correlated with cell proliferation as MSE has been reported to increase cell proliferation [4].

Hypoglycemic activity due to stimulation of insulin production and glucose utilization has been reported from green fruit of plantain banana and fibers from fruit were found to increase glucogenesis in the liver and lowered fasting blood glucose. *Musa sapientum* has been reported to contain β-sitosterols, leucocyanidin, sryngin, quercetin, sterylglycosides, aminoacids etc. β-sitosterols and dimethoxy derivative of leucocyanidin, leucocyanidin 3-O-beta-D-galactosyl cellobioside, have demonstrated hypoglycemic effect in experimental animal [16]. Flavonoids are most commonly known for their antiulcer activity, lipid lowering activity, anti-inflammatory activity, antioxidant activity, antimicrobial activity [31] and anti-hyperglycaemic [32] activities. HPTLC fingerprinting of MSE demonstrated eight active constituents where one of the constituents may be leucocyanidin, as reported earlier by Prabha and associates [17], who have compared the constituent with authenticated standard leucocyanidin. Alkaloids have been reported to show anti-diabetic and anti-inflammatory, antioxidant activity [33] while saponins showed anti-diabetic effects [34].

## Conclusion

The presence of many active constituents like flavonoids, saponins, glycosides and alkaloids in MSE, having prominent anti-hyperglycemic, antioxidants and ulcer healing properties, may contribute in healing of chronic diabetic gastric ulcer possibly by decreasing gastric mucosal cytokines, IL-1β and TNF-α and enhancing, growth factor, TGF-α. The possible control of blood sugar level by MSE has additionally reversed the adverse effects of diabetes on ulceration and cytokines. PTX, an inhibitor of TNF-α_,_ a prominent inflammatory marker, and MSE showed better healing effect in DR-AA rat than omeprazole or insulin. Thus, MSE could be a better choice in diabetes with concurrent peptic ulcer.

## Abbreviations

PCR: Polymerase chain reaction; TNF-α: Tumor necrosis factor-α; IL-1β: Interleukin-1β; TGF-α: Transforming growth factor-α; AA: Acetic acid; GU: Gastric ulcer; DR: Diabetic rat; MSE: *Musa sapientum* extract; OMZ: Omeprazole; INS: Insulin; PTX: Pentoxyphylline; HPTLC: High-performance thin-layer chromatography, NR, Normal rat; SOD: Superoxide dismutase; CAT: Catalase; GSH: Glutathione peroxidase; LPO: Lipid peroxidation; NO: Nitric oxide; IGF-1: Insulin like growth factor-1; VEGF: Vascular endothelial growth factor; TGF-α: Transforming growth factor-α; CMC: Carboxyl methyl cellulose; STZ: Streptozotocin; BGL: Blood glucose level; RNA: Ribonucleic acid; DEPC: Diethylpyrocarbonate; cDNA: Complementary deoxyribonucleic acid; DTT: Dithiothreitol; dNTP: Deoxyribonucleotide triphosphate; DNA: Deoxyribonucleic acid; bp: Base pair; UV: Ultraviolet–visible; UI: Ulcer index; NIDDM: Non-insulin dependent diabetes mellitus.

## Competing interests

We declare that we have no conflict of interests.

## Authors’ contributions

MK: performed the study as his PhD work; MKG: literature search, data acquisition, data analysis and prepared the manuscript; AS: helped in the preparation of the extracts and experimental studies; RKG: supervised and evaluated the data and review the manuscript for publication. All authors read and approved the final manuscript.

## Pre-publication history

The pre-publication history for this paper can be accessed here:

http://www.biomedcentral.com/1472-6882/13/305/prepub
